# Suspended Metasurface for Broadband High-Efficiency Vortex Beam Generation

**DOI:** 10.3390/ma15030707

**Published:** 2022-01-18

**Authors:** Luyi Wang, Hongyu Shi, Jianjia Yi, Liang Dong, Haiwen Liu, Anxue Zhang, Zhuo Xu

**Affiliations:** 1 Shaanxi Key Laboratory of Deep Space Exploration Intelligent Information Technology, Xi’an Jiaotong University, Xi’an 710049, China; bigcrash@stu.xjtu.edu.cn (L.W.); hwliu1975@xjtu.edu.cn (H.L.); 2 MOE Key Laboratory for Multifunctional Materials and Structures, Xi’an Jiaotong University, Xi’an 710049, China; 3 School of Information and Communications Engineering, Xi’an Jiaotong University, Xi’an 710049, China; jianjia.yi@mail.xjtu.edu.cn (J.Y.); anxuezhang@xjtu.edu.cn (A.Z.); 4 Yunnan Observatories, Chinese Academy of Sciences, Kunming 650216, China; dongliang@ynao.ac.cn; 5 Electronic Materials Research Laboratory, Key Laboratory of the Ministry of Education, Xi’an Jiaotong University, Xi’an 710049, China; xuzhuo@xjtu.edu.cn

**Keywords:** suspended metasurface, vortex beam, orbital angular momentum, broadband, high-efficiency

## Abstract

Electromagnetic (EM) waves carrying orbital angular momentum (OAM) exhibit phase vortex and amplitude singularity. Broadband OAM generation with high efficiency is highly desired with suggested applications such as broadband imaging and communications. In this paper, suspended metasurface structure achieving low-Q factor is proposed to realize broadband phase control and excellent reflection efficiency. Broadband vortex beam generation with OAM order of 1 and 2 are realized using the proposed suspended structure. Furthermore, by analyzing different metasurface aperture phase distribution schemes, the efficiency of the OAM generator is maximally achieved. The designs are validated by simulation and measurement. The proposed OAM generators work across 4–10 GHz with efficiency higher than 82%. This design provides a route to broadband metasurface realization and high efficiency OAM generation.

## 1. Introduction

Vortex beams refer to the OAM carrying EM waves with phase vortex and amplitude singularity in the propagating direction. The OAM, along with spin angular momentum (SAM), which manifests as the polarization of the EM waves and is already well exploited, constitute the whole angular momentum (AM) of EM waves [1]. OAM offers a new degree of freedom in EM waves and it has been researched extensively from the generation to the applications in recent years. The potential applications in imaging [2,3,4] and communication [5,6,7,8] are proposed along with particle manipulating [9,10], spin detection [11], and so on.

The foundation of these applications is the vortex beam source. Various methods have been proposed to generate vortex beams, such as antenna arrays [6,12], spiral phase plates [13,14], q-plates [15,16], holographic diffraction gratings [17], reflect arrays [18,19,20], metasurfaces [21,22,23,24,25,26,27,28,29], and so on. Almost all methods, apart from the antenna arrays approach, are centered on introducing OAM to the impinging wave, which involves applying the vortex phase to the incident waves, which can be expressed by the term exp(*ilΦ*), where *Φ* is the azimuthal angle and *l* is the OAM order. When imparting the vortex phase to the incident waves, discrete and continuous metasurface aperture phase distribution schemes are feasible solutions. However, the vortex generation efficiencies related to these phase distribution schemes are, to the best of the author’s knowledge, not studied before.

The bandwidth of the vortex beams is also of critical interest in potential applications such as broadband imaging [2]. Literature on broadband vortex generation is mainly based on the metasurface approach, Pancharatnam–Berry (PB) metasurface in particular [24,25,26,27,28,29]. Other methods such as hybrid helix array also exist [30]. In this paper, metasurface structure with intrinsic low-Q factor is realized with the suspended structure that can suppress the magnetic coupling between metasurface and ground plane to further enhance the broadband characteristic of the metasurface. In addition, a suspended structure is conducive to a lightweight design and can further lower the fabrication cost.

In this paper, the proposed suspended metasurface unit cells can precisely control the phase of the reflected circularly polarized (CP) wave across broadband (4–10 GHz) with excellent efficiency (over 99%). Furthermore, by analyzing the discrete and continuous metasurface aperture phase distribution schemes, the efficiency of the OAM generator is maximally achieved. Suspended metasurfaces generating broadband vortex beam with OAM order of 1 and 2 across 4–10 GHz with efficiency higher than 82% were designed and fabricated. The simulation results agree well with the measurement. Our design presents a route for broadband metasurface realization and high efficiency vortex beam generation.

## 2. Metasurface Design

**Figure 1:**
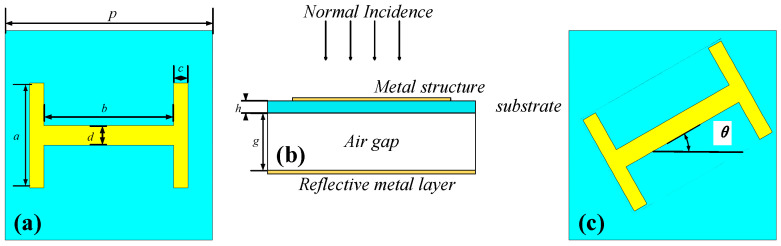
The model of the proposed PB unit cell: (**a**) Front view. (**b**) Side view. (**c**) Rotated view.

The pattern of the proposed metasurface unit cell is shown in Figure 1, where the blue represents substrate F4B-TM2 with $\varepsilon_{r}\;$ = 6.15 and tanδ = 0.0025. The yellow represents copper with a thickness of 0.035 mm. The unit cell is composed of I shaped metal printed on a thin layer of substrate, which is suspended from the reflective metal layer at the bottom by an air gap. The detailed geometry of the design is as follows: *p* = 15.67 mm, *a* = 7.84 mm, *b* = 9.79 mm, *c* = 1.08 mm, *d* = 1.47 mm, *h* = 0.51 mm, *g* = 7.6 mm. The height and periodicity of the unit cell is 8.11 mm (0.19 × λ) and 15.67 mm (0.36 × λ) respectively, where λ is the wavelength at central frequency 7 GHz.

For the PB metasurface, the phase of the reflected wave can be controlled by the different rotation angles *θ* (anticlockwise) of the I shaped structure. For an incident CP wave, the polarization of the reflected wave is preserved, and |2*θ*| abrupt phase change compared to the unrotated situation is added. In particular, the abrupt phase change is 2*θ* for a right-handed CP (RHCP) wave and −2*θ* for a left-handed CP (LHCP) wave [31]. That is, the reflection phase gradient is opposite under different incident helicities. The air gap brought by the suspended structure reduces the magnetic coupling between metasurface and reflective plane, which suppressed the Q-factor of magnetic resonance and results in the phase control in a broadband [32].

**Figure 2:**
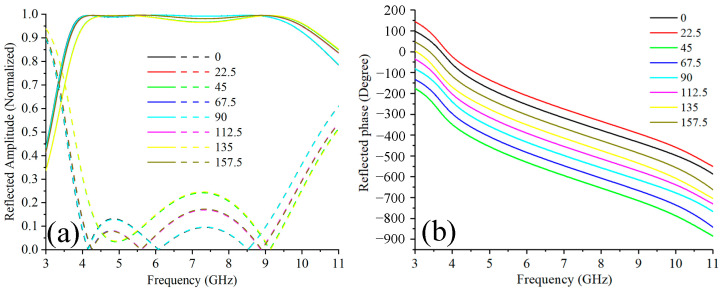
The simulation results of the proposed PB unit cell under different rotation angles: (**a**) Reflection amplitude. (**b**) Reflection phase.

The properties of the proposed unit cell were acquired by simulation using commercial software CST Microwave Studio (Version 2016, Computer Simulation Technology GmbH, Darmstadt, Germany). The co-polarization and cross-polarization amplitude of the reflected wave under LHCP illumination were depicted in Figure 2a in solid and dashed line, respectively. Eight curves representing unit cells with eight selected rotation angles are shown. In all these cases, from 3.9 GHz to 10.6 GHz, the amplitude of the co-polarization component remains higher than 0.9. It should be noted not all the curves overlapped and slight discrepancies between different rotation angles exist. This can be explained by the change of the resonance mode of the unit cell under different angles, however, in all these cases the characteristics of the unit cells are similar. The efficiency of the unit cell can be calculated by the ratio of the co-polarization energy to the total reflected energy. From 3.9 GHz to 10.6 GHz, the efficiency is higher than 83%. Specifically, the efficiency is higher than 94% from 4.1 GHz to 10 GHz. High conversion efficiency ensures minimum cross-polarization component was reflected, a side effect that not only degenerates the efficiency of the OAM generating metasurface but also affects the purity of the reflected vortex beams. Figure 2b shows the reflected phase of the co-polarization component. Eight rotation angles correspond to eight phase curves which cover the 360° phase change with a step of 45° in the operating band, which validated the PB theory that the phase of the reflected wave changed 2*θ* compared to the unrotated situation. Notably, if the handness of the incident wave changes, the phase gradient of the reflected wave will change, too. It should also be noted that a parallel phase response relation between different rotation angles is observed within the whole operating band, which is crucial for the broadband vortex phase distribution design. To sum up, the suspended unit cell we proposed can realize 360-degree precise phase control of the reflected wave with high efficiency across broadband. This paves the way for the design of metasurface for broadband OAM generation and other applications.

**Figure 3:**
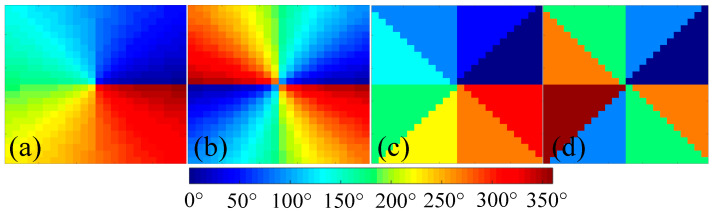
The front view of the continuous vortex phase distribution schemes for generating beams carrying OAM of different orders: (**a**) *l* = 1. (**b**) *l* = 2. The discrete vortex phase distribution schemes for generating beams carrying OAM of different orders: (**c**) *l* = 1. (**d**) *l* = 2.

To impart OAM to an impinging wave, unit cells with uniform reflection amplitude but different reflection phase should be placed on the corresponding area of the metasurface according to the vortex phase distribution denoted by the term exp(*ilΦ*). To be specific, the vortex beam with OAM order of *l* experiences an azimuthal phase change of |*l*| × 360°. In this paper, we compared and analyzed the discrete and continuous metasurface aperture phase distribution schemes and their respective efficiency for OAM generation. As illustrated in Figure 3a,b, continuous metasurface aperture phase distributions for the generation of OAM of orders 1 and 2 strictly complied with the term exp(*ilΦ*), with every unit cell manifesting different phases, whereas, in Figure 3c,d, discrete metasurface aperture phase distributions sectioned the aperture into eight sectors, with each sector manifesting the same phases.

**Figure 4:**
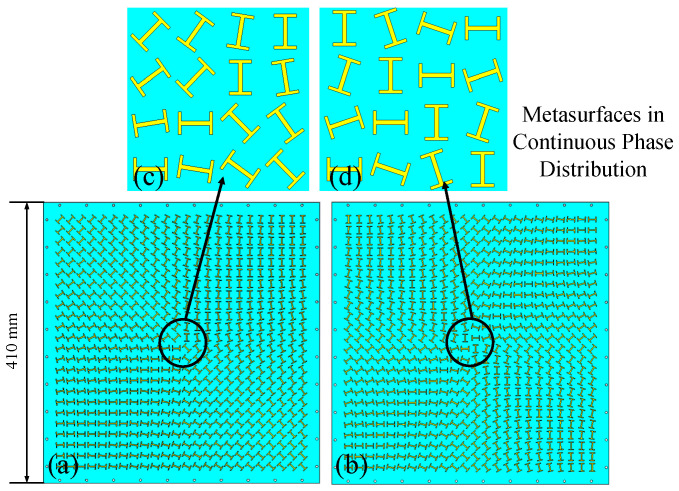
The simulation model of the proposed metasurfaces in continuous phase distribution: (**a**) Front view of the metasurface generating OAM order of 1. (**b**) Front view of the metasurface generating OAM order of 2. (**c**) Detailed view of the metasurface generating OAM order of 1. (**d**) Detailed view of the metasurface generating OAM order of 2.

In our design, the phase distribution is discretized into 24 × 24 elements and each corresponds to the required reflection phase of the unit cell. Firstly, according to the designed reflection phase distribution schemes, the rotation angles of the unit cells are calculated and the unit cells are arranged accordingly to form metasurfaces consisting of 24 × 24 elements, as shown in Figure 4. For brevity, we only show the models with continuous metasurface aperture phase distributions. The margin of the metasurfaces is punched with a series of holes, which are used for supporting the metasurface and the reflective metal plate at the bottom. The reflective metal plate has the same dimension as the metasurface and is omitted in Figure 4. The proposed structures have an overall size of 410 mm × 410 mm with a thickness of 12 mm. The models were simulated by CST Microwave studio using broadband RHCP Gaussian beam as excitation. The minimum beam radius of the incident beam is 120 mm, thus ensuring the majority of the incident energy is reflected. The phase profile of the Gaussian beam at the focal plane, where the metasurfaces were placed, is close to that of a plane wave.

## 3. Simulated and Measurement Results

The efficiency of the metasurface is first analyzed. The efficiency of the OAM generating metasurface is defined as the power ratio of the co-polarization component of the reflected wave (the generated vortex beam) to the total incident energy. The efficiencies for OAM generation with discrete and continuous schemes are analyzed and given in Table 1. The metasurface efficiency can also be expressed by the formula (*I–T–L–C)/I*, where *I* represents the total incident energy, *T* represents the transmitted energy (i.e., the diffracted energy), *L* represents the energy loss in the metasurface, *C* represents the energy of the cross-polarization component. From the formula, we can see cross-polarization component suppression is a critical aspect of realizing high efficiency.

For metasurface with continuous aperture phase distribution reflecting beams with OAM order of 1, the efficiency is higher than 92% across the band. Compared with other frequencies, efficiencies at 7, 8 and 10 GHz are lower, which are caused by the relatively low efficiency of the metasurface at these frequencies and are in accord with the far-field pattern results. The efficiencies are generally lower for the OAM order of 2 case. This is caused by the higher cross-polarization component of the reflected wave, however, high efficiency can still be achieved, compared with other works such as [23,24]. For metasurface with discrete aperture phase distribution, the efficiency is slightly lower than the continuous distribution case, except at some frequency point. This slightly inferior performance can be attributed to the phase error induced by the aperture section. In this paper, therefore, we utilized the continuous aperture phase distribution to further conduct the research. The discrete aperture phase distribution, however, is still a reasonable choice when the phase change characteristic of the unit cell is not continuous or not easily attainable.

**Figure 5:**
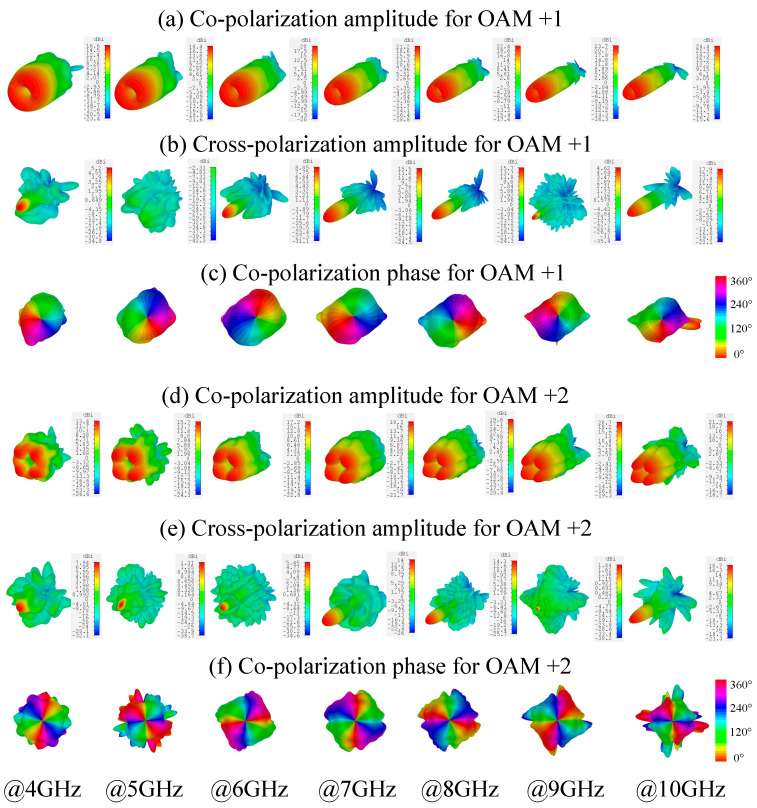
The simulated far-field pattern of the reflected beams: (**a**) Co-polarization and (**b**) Cross-polarization amplitude for OAM + 1. (**c**) Co-polarization phase for OAM + 1. (**d**) Co-polarization and (**e**) Cross-polarization amplitude for OAM + 2. (**f**) Co-polarization phase for OAM + 2.

The simulated broadband far-field patterns of the reflected wave under RHCP illumination are presented in Figure 5. Figure 5a shows the RHCP component (co-polarization) of the reflected beam from 4 to 10 GHz with a step-size of 1 GHz. The generated vortex beams of OAM order of 1 exhibit an amplitude null at the center across the broadband. The divergence angle and the directivity of the beams increase with the frequency. The LHCP component (cross-polarization) of the reflected beam, as shown in Figure 5b, are caused by the cross-polarization reflection of the metasurface. It can be observed at frequencies such as 7, 8 and 10 GHz that the amplitude of the reflected cross-polarization is relatively large. This is caused by the relatively low conversion efficiency of the metasurface at these frequencies and is in accord with the characteristics of the unit cell. Although the relatively large amplitude, the cross-polarization is at least 5.7 dB (at 7 GHz) lower than the vortex beam and it is demonstrated that the efficiency is still high. The phase profiles of the broadband vortex beam are shown in Figure 5c, where at all the frequencies clear vortex phase distribution of OAM order of 1 can be observed. The phase singularity coincides with the amplitude null, indicating the broadband vortex beam generation characteristic of the proposed metasurface. Outside the working band, however, the performance of the metasurface deteriorates quickly. Moreover, it is reasonable if the metasurface is under LHCP illumination, the reflected wave will be a similar vortex beam with OAM order of −1.

Figure 5d–f depicts the co-polarization far-field patterns, the cross-polarization far-field patterns and the phase profiles of the broadband vortex beam respectively for the OAM order of 2 case. Similarly, amplitude null at the center across the broadband can be observed. Furthermore, 4π phase accumulations along a full circular path around the beam null across the whole band indicate the generation of OAM order of 2.

**Figure 6:**
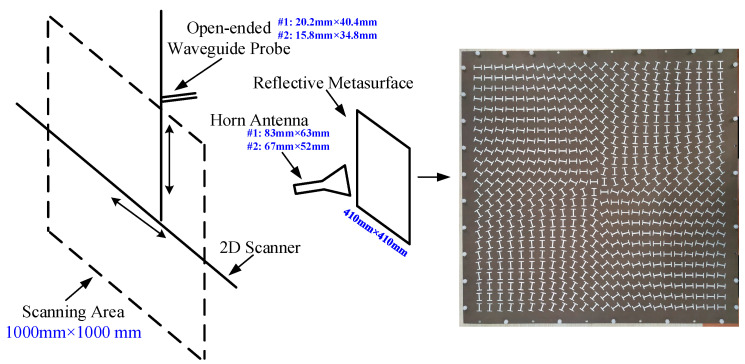
The fabricated metasurface and the near field measurement settings.

The proposed metasurfaces were fabricated using PCB processing and a 4 mm thick aluminum reflective metal plate with the same geometry was cut to match the metasurface. The dimensions of the fabricated metasurface are 410 mm × 410 mm in width and length, with a thickness of 0.51 mm. At the perimeter of both the metasurface and the reflective metal plate, 40 evenly spaced drill holes were placed to allow the nylon screws to support the suspended structure. Furthermore, rigid foam is inserted under the substrate to enhance the stability of the metasurface and to ensure the air gap width. In total, the structure has a thickness of 12 mm. The fabricated metasurface and the schema of the near field measurement settings are shown in Figure 6.

**Figure 7:**
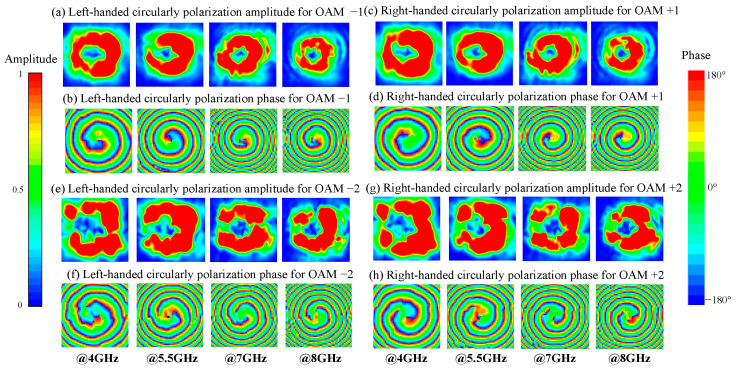
Measured near field distribution of the reflected beams: (**a**) LHCP amplitude and (**b**) phase for OAM − 1. (**c**) RHCP amplitude and (**d**) phase for OAM + 1. (**e**) LHCP amplitude and (**f**) phase for OAM − 2. (**g**) RHCP amplitude and (**h**) phase for OAM + 2.

The near field of the reflected wave was measured in an anechoic chamber using the settings depicted in Figure 6. The distance between the horn antenna and the metasurface is 100 mm, the distance between the metasurface and the probe is 500 mm. Due to the limited working bandwidth, two horn antennas were used respectively as the excitations, they are used separately and together they generate linearly polarized waves from 4–8 GHz. For the same reason, two open-ended rectangular waveguide probes working at corresponding frequencies were used for receiving the reflected OAM carrying beams. The vertically and horizontally polarized electric fields were measured respectively by rotating the receiving probe, then the data were post-processed to show the LHCP and RHCP component. The scanning area is 1000 mm × 1000 mm. Here it should be noted that a linearly polarized wave can be decomposed into RHCP and LHCP with the same intensity. Thus ideally, the metasurfaces would reflect the incident linearly polarized waves into LHCP vortex beam of OAM order −1/−2 and RHCP vortex beam of OAM order +1/+2. The measured near field of the reflected wave were illustrated in Figure 7.

Figure 7 shows the near field of the reflected wave at 4 GHz, 5.5 GHz, 7 GHz and 8 GHz respectively. The plot size is 0.25 × 0.25 m^2^ since the margin of the scanned area is with negligible field distribution. In Figure 7a,c,e,g, clear donut-shaped amplitude distributions were seen across the broadband. In Figure 7b,d,f,h, phase distribution of OAM order −1, +1, −2, +2 can be clearly seen. The near field scanning results verified the generation of LHCP with OAM order −1/−2 and RHCP with OAM order +1/+2 across the frequency band from 4–8 GHz.

The OAM purities of the reflected beams are also investigated using the method in [28,29] and listed in Table 2. The desired OAM modes account for at least 78.6% of the energy of the reflected beams. From the calculated OAM purities, we conclude that the unwanted OAM order spectrum is sufficiently low and have minor affection to the desired OAM mode.

**Figure 8:**
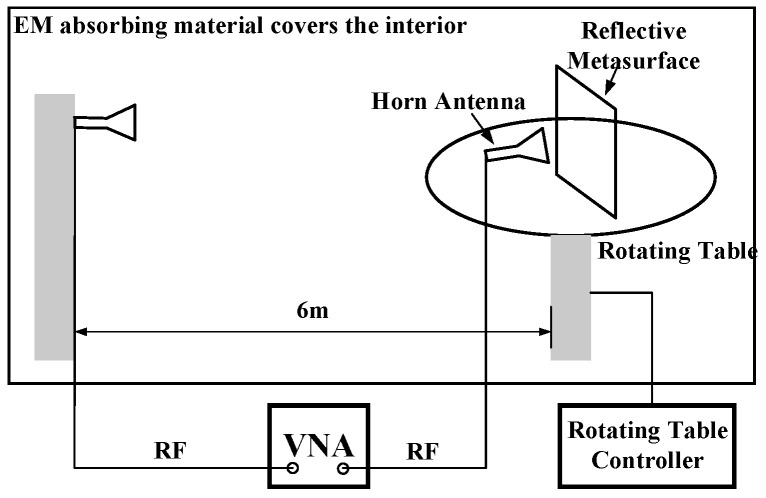
The far field measurement settings.

In Figure 8 we illustrate the far field measurement setup schematic. The far field pattern was measured in an anechoic chamber using a single probe measurement system. The metasurface is placed on the rotating table while the standard horn antenna is attached to the stationary pole 6 m away.

**Figure 9:**
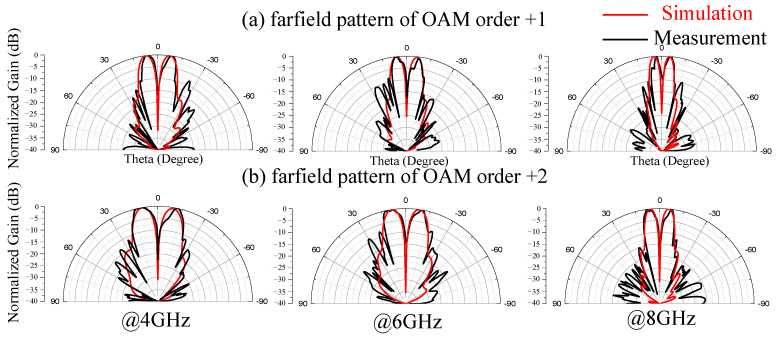
Measured and simulated far-field pattern of the reflected RHCP vortex beam: (**a**) OAM order + 1. (**b**) OAM order + 2.

The far-field patterns were measured, processed and shown in Figure 9 in black lines. For brevity, we only show the far-field of the reflected RHCP vortex beam at 4 GHz, 6 GHz and 8 GHz. The corresponding simulated results is also shown in Figure 8 in red lines for comparison. The simulated results and measurement results agree well with each other. It can be seen from Figure 9 that the vortex beams show clear amplitude nulls at the propagating direction.

Finally, to exhibit the advantages of our design, we compare our work with other broadband OAM generation schemes in Table 3. The OAM bandwidth and generation efficiency in our work is further extended compared with other works except in [25,29]. However, [25] utilized a multilayer structure and [29] suffer from high in-band cross-polarization level. The air-suspended structure in our work is more cost-effective by reducing the substrate layer thickness and offer a degree of freedom in PB metasurface design.

## 4. Conclusions

In conclusion, suspended Pancharatnam–Berry metasurfaces were designed, fabricated for high efficiency broadband vortex beam generation. Discrete and continuous metasurface aperture phase distribution schemes and their respective efficiency for OAM generation is analyzed. The designed metasurfaces convert the incident wave into LHCP vortex beams of OAM order −1/−2 and RHCP vortex beams of OAM order +1/+2 across the frequency band from 4–10 GHz with efficiency over 82%. This design presents a solution for broadband vortex beam generation and can be useful in possible broadband vortex beam applications.

## Figures and Tables

**Table 1:** Simulated efficiency of the metasurfaces under different aperture phase distribution.

		Frequency (GHz)	4	5	6	7	8	9	10
OAM order 1	Continuous	Efficiency	94.9%	97.4%	96.3%	92%	92.4%	97.7%	92.6%
	Discrete		96.0%	94.8%	95.6%	92.2%	91.4%	95.7%	90.6%
OAM order 2	Continuous	Efficiency	84.3%	91.3%	92.6%	91.2%	91.5%	94.5%	87.4%
	Discrete		83.6%	89.5%	91.0%	90.6%	91.1%	92.9%	86.5%

**Table 2:** Measured OAM purities of the reflected beams.

	Frequency (GHz)	4	5.5	7	8
OAM order + 1	Mode Purity	83.0%	88.3%	82.4%	84.5%
OAM order − 1		84.5%	84.5%	85.2%	83.8%
OAM order + 2	Mode Purity	78.9%	81.6%	80.5%	81.7%
OAM order − 2		80.9%	79.2%	78.6%	80.2%

**Table 3:** Comparison of broadband OAM generation schemes in literature.

Ref	Frequency Range (GHz)	Relative BandWidth (%)	OAM Generation Efficiency (%)	Layer Number	Layer Thickness (mm)
[24]	12–18	40	75.76	1	3
[25]	6.95–18	88.5	N/A	2	3.3
[26]	59–70	17	64	1	0.63
[27]	18–28	43.5	65	1	2.4
[28]	8.55–19.95	80	N/A	1	3
[29]	6–19.7	107.2	N/A	1	4
[30]	8.1–13	46.5	65	1	N/A
**Our Work**	**4**–**10**	**85.7**	**82**	**1**	**0.51**

## Data Availability

The data presented in this study are openly available.
